# A New Method of Treatment of Temporomandibular Joint Ankylosis with Osteodistraction Using the Sh-Device: A Case Report

**Published:** 2018-01

**Authors:** Gholamreza Shirani, Mahnaz Arshad, Xaniar Mahmoudi

**Affiliations:** 1 Assistant Professor, Dental Research Center, Dentistry Research Institute, Tehran University of Medical Sciences, Tehran, Iran; Department of Oral and Maxillofacial Surgery, School of Dentistry, Tehran University of Medical Sciences, Tehran, Iran; 2 Assistant Professor, Dental Research Center, Dentistry Research Institute, Tehran University of Medical Sciences, Tehran, Iran; Department of Prosthodontics, School of Dentistry, International Campus, Tehran University of Medical Sciences, Tehran, Iran; 3 Dentistry Student, School of Dentistry, International Campus, Tehran University of Medical Sciences, Tehran, Iran

**Keywords:** Temporomandibular Ankylosis, Distraction Osteogenesis, Bone Lengthening, Tissue Expansion, Treatment Outcome

## Abstract

This case report presents a 16-year-old boy with bilateral temporomandibular joint (TMJ) bony ankylosis due to trauma. The patient had undergone several unsuccessful therapeutic surgeries and was experiencing reduced mouth opening, difficulty in eating and speaking, poor oral hygiene, snoring, and depression. Bilateral gap arthroplasty and distraction osteogenesis (DO) were performed. After the surgery, we were able to move the osteodistractors forward and prevent the upward and backward movement of the proximal mandibular segment with the use of our custom-made Sh-device, which allowed bone growth and soft-tissue matching. The mandibular deficiency was treated, and the patient’s sleep quality significantly improved after three months. The physical, orthodontic and speech therapies were continued. The facial asymmetry, difficulty in sleeping, eating and speaking, and low self-esteem were completely resolved. At the 8-year follow-up, the patient’s condition was satisfactory. The Sh-device can be used as a contemporary treatment modality for TMJ ankylosis.

## INTRODUCTION

Temporomandibular joint (TMJ) ankylosis refers to the adhesion between the mandibular condyle and the glenoid fossa, maxilla, zygoma or the base of the skull [1]. This adhesion may be fibrous or bony [2]. TMJ ankylosis is usually associated with trauma, systemic infection, or systemic diseases such as ankylosing spondylitis, rheumatoid arthritis, and psoriasis. It can also be congenital or secondary to severe rheumatoid arthritis or tumors in the TMJ region or may develop as a result of orthognathic surgery [1,3,4]. In TMJ ankylosis, mouth opening becomes limited and the patient experiences problems associated with facial growth, chewing, digestion, speech, aesthetics and oral hygiene maintenance [5]. The mandibular deficiency and craniofacial abnormalities and complications can lead to obstructive sleep apnea (OSA) [6,7]. The incidence of TMJ ankylosis has decreased because of the availability of advanced methods for the treatment of condylar fractures and the use of antibiotics for the treatment of infections; however, this condition is still common in some deprived parts of the world [6]. A minimally invasive treatment method with the highest rate of success must be employed for the management of TMJ ankylosis.

This condition is a serious challenge that poses technical difficulties during surgery, and it may recur [8]. Different surgical techniques can be used to correct the TMJ ankylosis such as mandibular advancement, genioplasty, distraction osteogenesis (DO) and orthognathic surgery [8]. DO is a gradual process that allows new bone formation in the surgically constructed gap. In DO, controlled forces of a low magnitude are applied, and the gradual stretching of the surrounding soft tissue stimulates tissue growth [9]. The involved tissues such as the skin, nervous tissue, muscles, tendons, ligaments and blood vessels adapt to this change. The ability of the surrounding soft tissues to withstand the exerted forces and their adaptation to the bone lengthening will guarantee the long-term success of this surgical procedure [9].

The distractors used in the maxillofacial region, to stimulate the growth of the deficient area, are either internal or external. The internal distractors are placed in the oral cavity. The bone-borne devices are attached to the bone; the tooth-borne distractors are attached to the teeth, while the hybrid distractors are attached to the bone and teeth. The external distractors are attached to the bone through the skin and are unidirectional, bidirectional, or multidirectional [10]. DO is indicated when orthognathic surgery is not the first treatment choice. This surgical method is recommended for various conditions such as facial asymmetry, midfacial hypoplasia, TMJ ankylosis, maxillary and mandibular deficiency, maxillofacial developmental disorders, palatal clefts, alveolar ridge augmentation, etc. [9–11].

In the patients with a history of gap arthroplasty, the proximal mandibular segment may move posteriorly since there is no posterior stop in the mandible, and this can complicate the use of DO in these patients [10]. Here, we report a new custom-made device for stabilizing the posterior mandibular segment and stimulating the anterior mandibular segment to grow forward during the DO.

## CASE REPORT

**Fig. 1:**
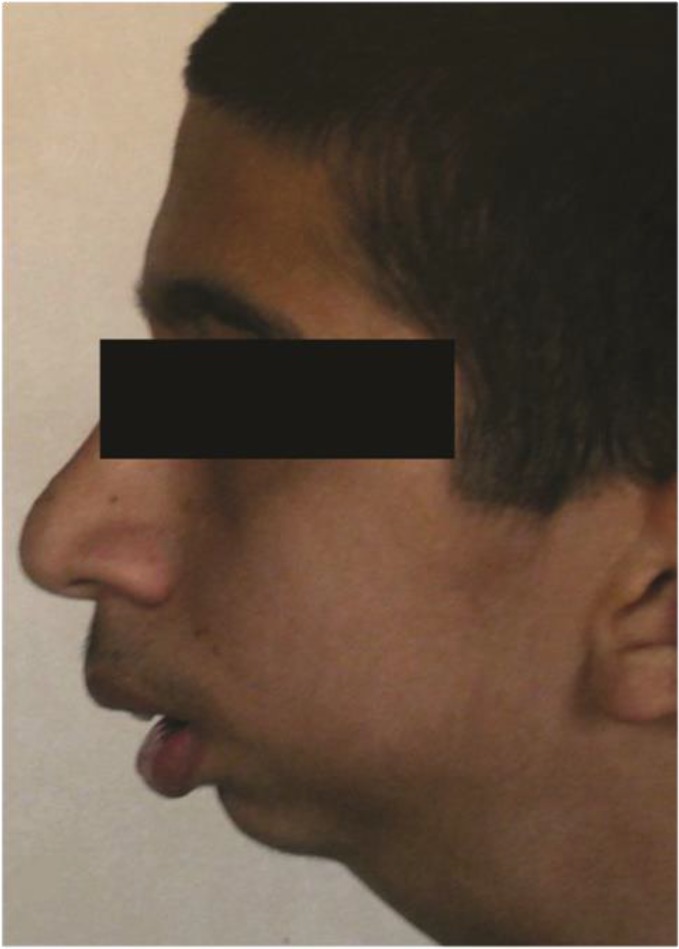
Preoperative left lateral view

A 16-year-old boy with bilateral TMJ ankylosis was referred to the oral and maxillofacial surgery department of Tehran University of Medical Sciences. The bilateral TMJ ankylosis had been induced due to trauma when the patient was 4 years old. He had undergone TMJ surgery twice, but the operations had been unsuccessful and the TMJ ankylosis relapsed (Fig. 1).

The clinical examination revealed no systemic diseases. The patient’s maximum incisal opening was 11mm. Because of the old injuries and unsuccessful operations, the mandibular deficiency had developed. The patient was experiencing sleeping disorders and mostly slept in a prone or a sitting position due to respiratory problems. The lateral and forward motions of the mandible were restricted. Based on the patient’s condition and the past surgical history, a two-step treatment was performed. First, the gap arthroplasty method was applied, and afterwards, DO was performed.

The gap arthroplasty surgery was followed by physiotherapy. Approximately 3 to 10 days after the surgery, the patient was put on a soft diet and began the jaw-opening exercises. Three to four weeks after the surgery, a normal diet was started. After the discharge, the patient was visited once a week to evaluate the changes.

**Fig. 2:**
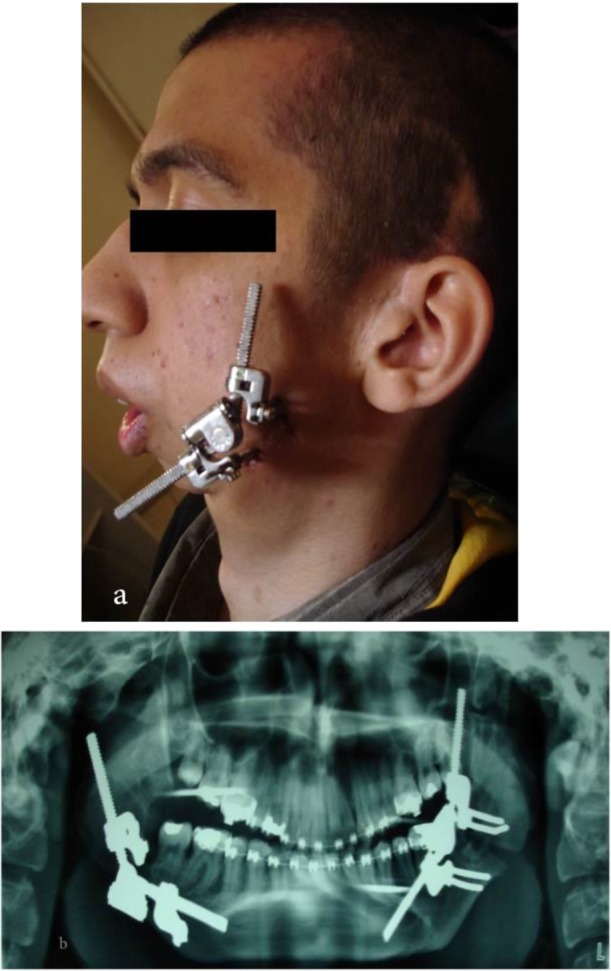
The distraction osteogenesis (DO) procedure. (a) Left lateral view. (b) Panoramic view

**Fig. 3:**
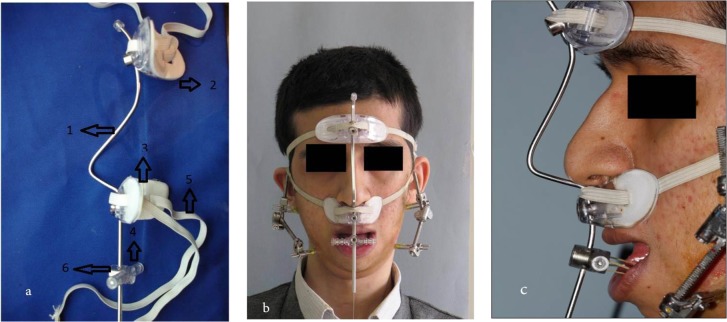
The Sh-device. (a) Left lateral view. (b) Frontal view of the patient wearing the Sh-device. (c) Lateral view of the patient wearing the Sh-device

The osteodistractor was installed four months later (Fig. 2). After the installation, since the condylar stop had been removed in this case, and there was a possibility of upward and backward shift of the proximal mandibular segment, we used a custom-made device to prevent these movements. The Sh-device has a curved metal rod and two oval stoppers that can be connected to two areas, one to the patient’s forehead and another to the upper lip (below the nose). The stoppers are made of a firm clear plastic; the inner stopper surface is covered with a layer of soft spongy tissue that provides comfort for the patient upon using the Sh-device (Fig. 3). The stoppers are attached to the back of the patient’s head by two elastic bands, which keep the device on the patient’s face. The elastic bands, which are attached to the orthodontic hooks on the mandibular premolars or molars, are connected to a small piece at the bottom of the lower stopper. The main pressure/traction of the Sh-device was applied on the distal mandibular segment, and the Sh-device prevented the proximal mandibular segment from moving upward and backward; therefore, the greatest displacement occurred at the distal mandibular segment. The mandible was driven forward at a rate of 0.5mm per day.

**Fig. 4:**
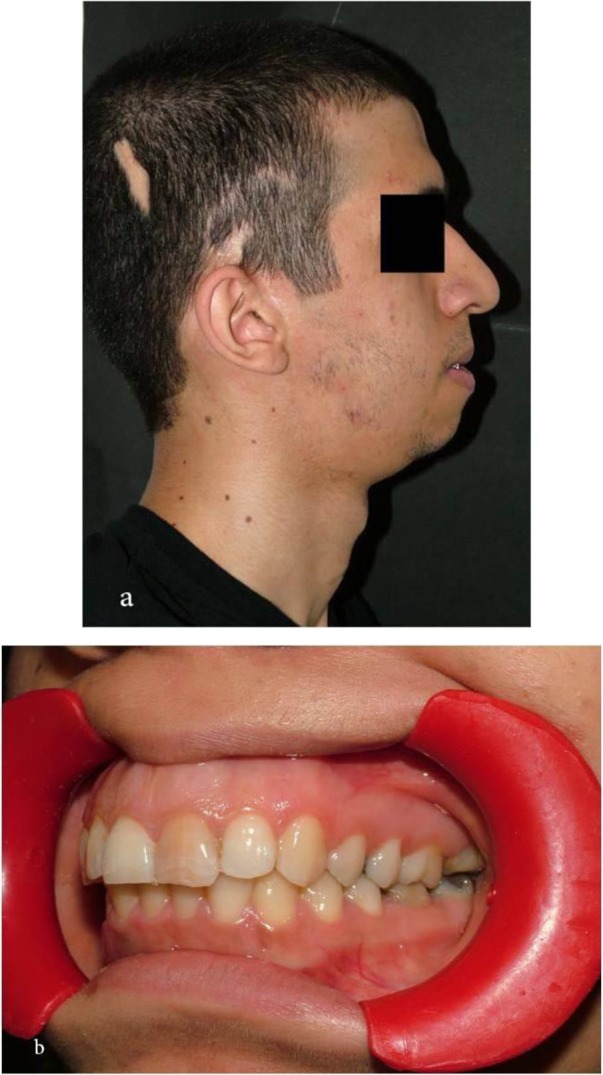
Final result of the treatment. (a) Right lateral view. (b) Left lateral view of the corrected dental occlusion

After one month, we separated the Sh-device, and the mandible moved about 15 to 20mm forwards. The patient’s breathing and sleeping improved. At the 8-year follow-up, the patient’s profile was satisfactory. The patient’s maximum incisal opening increased to 38mm (Fig. 4).

## DISCUSSION

Trauma is the most common reason for TMJ ankylosis. The prevalence of this condition is 13% to 100% [1]. TMJ ankylosis is a condition in which there is a connection between the mandibular condyle and the glenoid fossa. In growing children, TMJ ankylosis has a negative effect on the growth of facial bones and can disfigure the mandible causing chin deficiency, facial asymmetry, midline shift, malocclusion and tooth impaction [12]. Also, dental caries and periodontal disease occur because of a poor oral hygiene due to a reduced maximum incisal opening. Furthermore, facial skeletal problems can cause airway obstruction, and most patients suffer from sleep apnea [1,12].

Postsurgical aims include providing an adequate mouth opening, preventing re-ankylosis and creating an acceptable postoperative facial appearance. Various surgical procedures are available to achieve these objectives, such as mandibular advancement, genioplasty, DO and orthognathic surgery [13]. DO is one of the most popular methods. One of the benefits of DO over orthognathic surgery is the dynamic increase in the mandibular length and height. Also, DO greatly decreases the recurrence of ankylosis [13]. There are other advantages for DO as well, such as a reduced amount of blood loss during the surgery and subsequently, fewer postoperative complications, reduced duration of the surgery, less need for bone harvesting during the surgery, and minimized bone resorption during the recovery time. These advantages also decrease the medical costs [14].

One of the disadvantages of DO is the long duration of the treatment and the need for the cooperation of patients and their families as they have to pay attention to some essential tips when activating the device. Also, after full recovery, the patients need to undergo a second operation to remove the device; all of which are time-consuming and can be difficult for the patients [14]. Faultfinders of DO believe that after the DO treatment, the changes in the mandibular position during the distraction are not fully controllable, and the risk of ankylosis recurrence increases in this method [15]. The other potential problems raised by the surgical treatment of ankylosis include local inflammation at the site of device installation, TMJ discomfort upon using the device, and temporary inferior alveolar nerve paresthesia and mild pain upon activating the device, which can be relieved by analgesics [16]. After the DO treatment, the Sh-device allowed a forward movement of the mandible. The Sh-device eliminated the need for surgical installation of the device. Also, this device is minimally invasive and easy to use. It allows the free movement of the distal mandibular segment and prevents the following upward and backward movement of the proximal mandibular segment. After the surgical treatment of ankylosis, the postoperative mandibular deviation and facial asymmetry often persist [17]. The midline shift and facial asymmetry can be easily managed by the Sh-device.

The success rate of DO increases if care is taken not to traumatize the muscles surrounding the bone. Direct bone-to-bone contact should be avoided during DO. For this purpose, an inter-positional graft can be used, which reduces the force applied to the reconstructed bone [18].

## CONCLUSION

TMJ ankylosis causes facial disfigurement and loss of jaw function. These problems, as well as the midline shift and facial asymmetry, can be easily managed by DO using the Sh-device.
